# Supplementary material for: Maladaptive Inflammatory Signaling in Old Mice Impairs Colonic Regeneration by Promoting a Sustained Fetal‐Like Epithelial State
Source: Aging Cell. 2026 Apr 20;25(5):e70495. doi: 10.1111/acel.70495 (PMC13093538; doi:10.1111/acel.70495)

Figure S1. Markers used for annotation of the colon epithelial and immune cell types with epithelial cell type refined annotations and differential expression

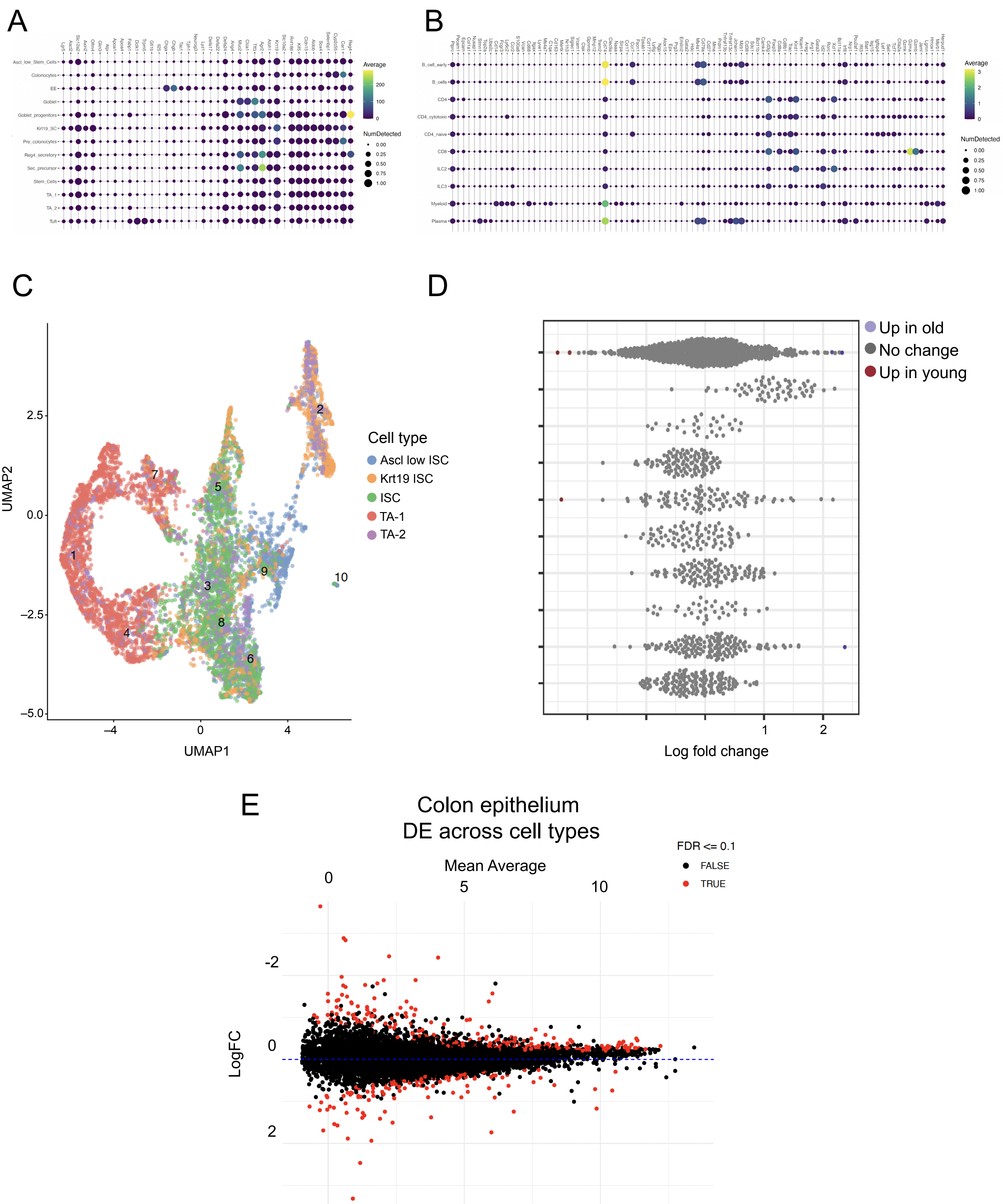

Supplemental Figure 2. Evidence for similar bacterial clearance rates in old vs. young colons

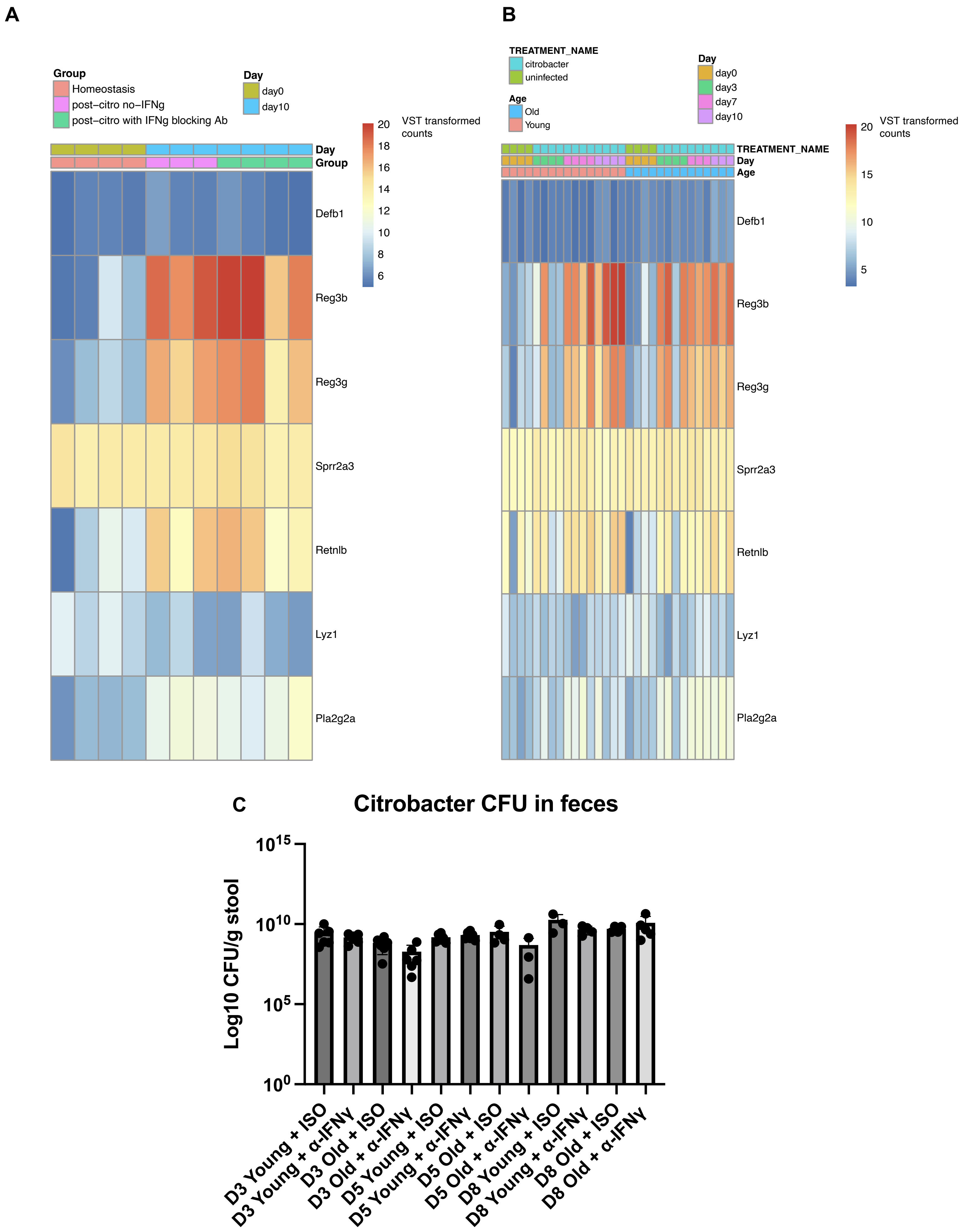

Figure S3. Analysis of Cleaved Caspase-3 (CC3) expression in young and aged colonic epithelium 7 days after C. rodentium infection

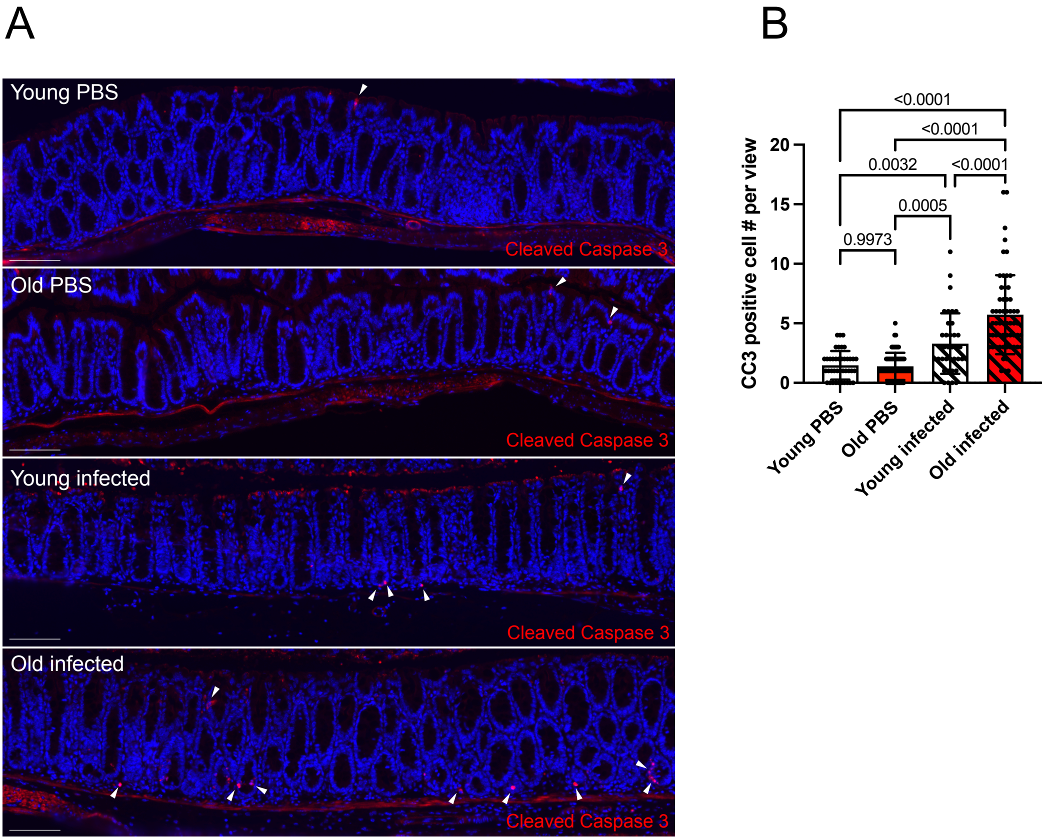

Scale Bar: 100 um

Figure S4. Aged colons exhibit higher epithelial damage during infection.

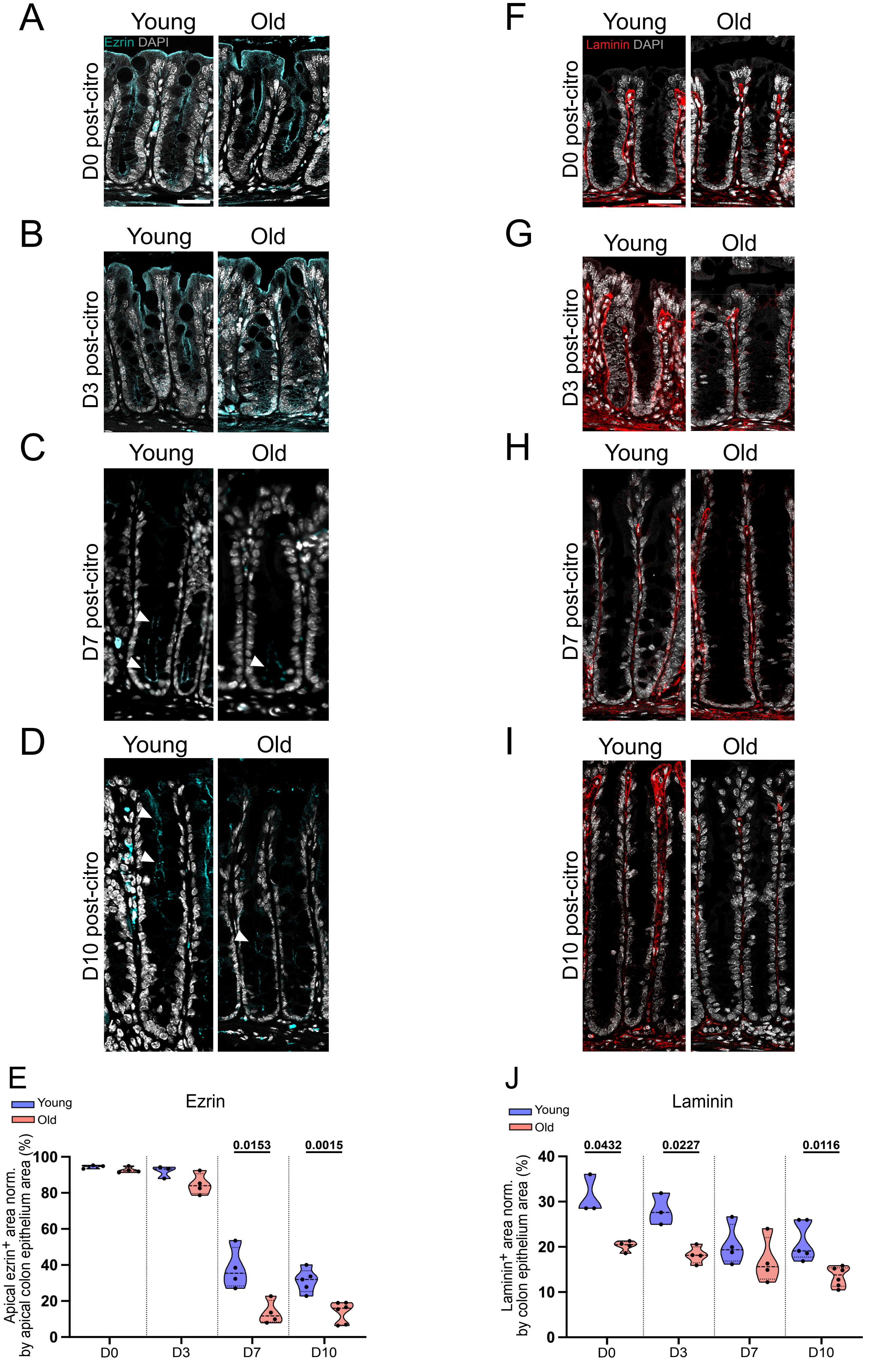

Figure S5. Aging remodels colonic immune composition and inflammatory effector programs during C. rodentium infection

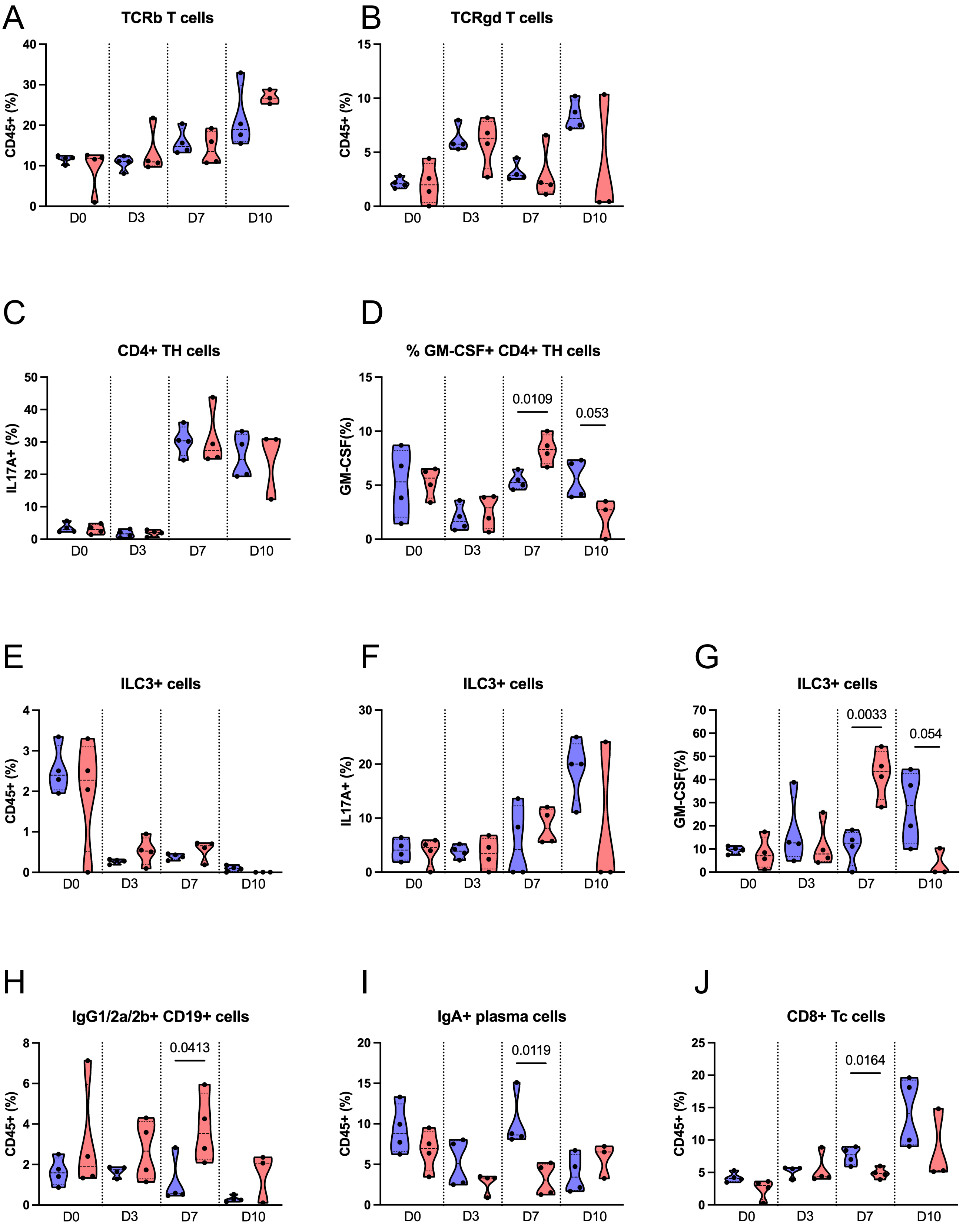

Figure S6. Decreased population of IL-22$^+$ CD4$^+$ T_H cells in the colon of old mice at day 10 post-*Citrobacter* infection.

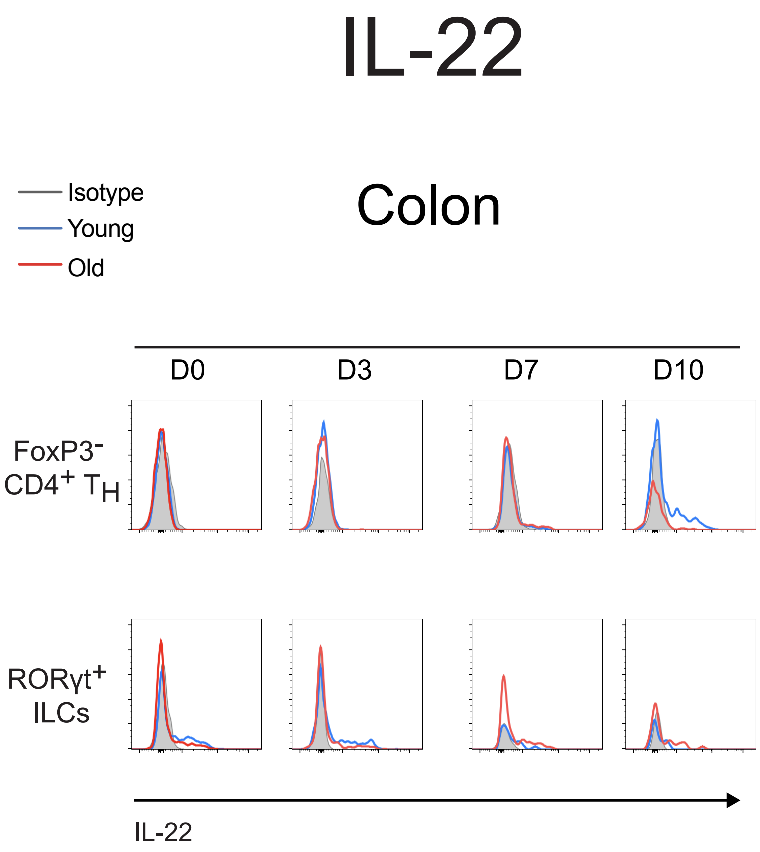

Figure S7. All clusters containing DE genes grouped based on their pattern of expression as a function of age.

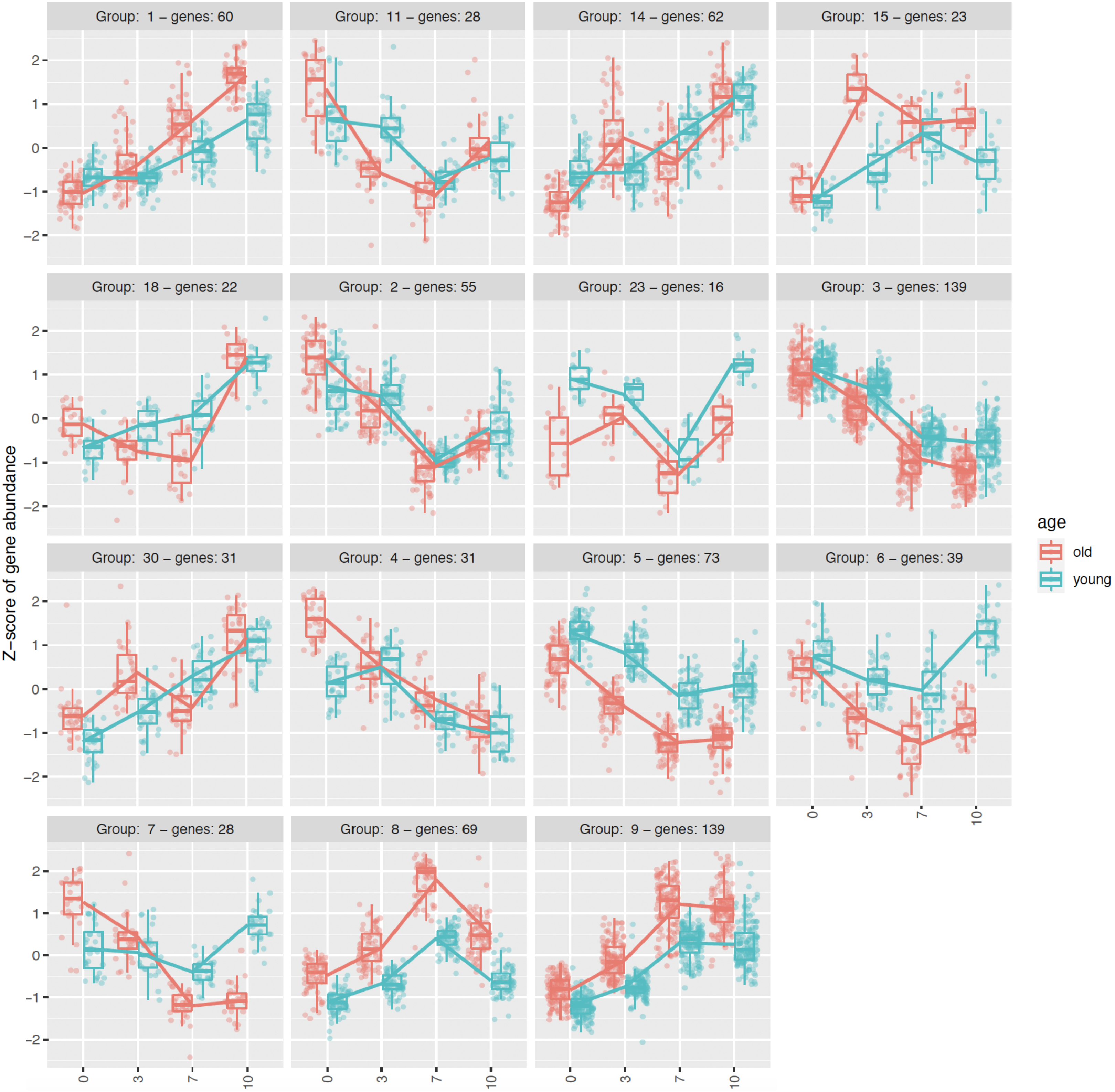

Figure S8. GO analysis of all clusters grouped based on their pattern of expression as a function of age.

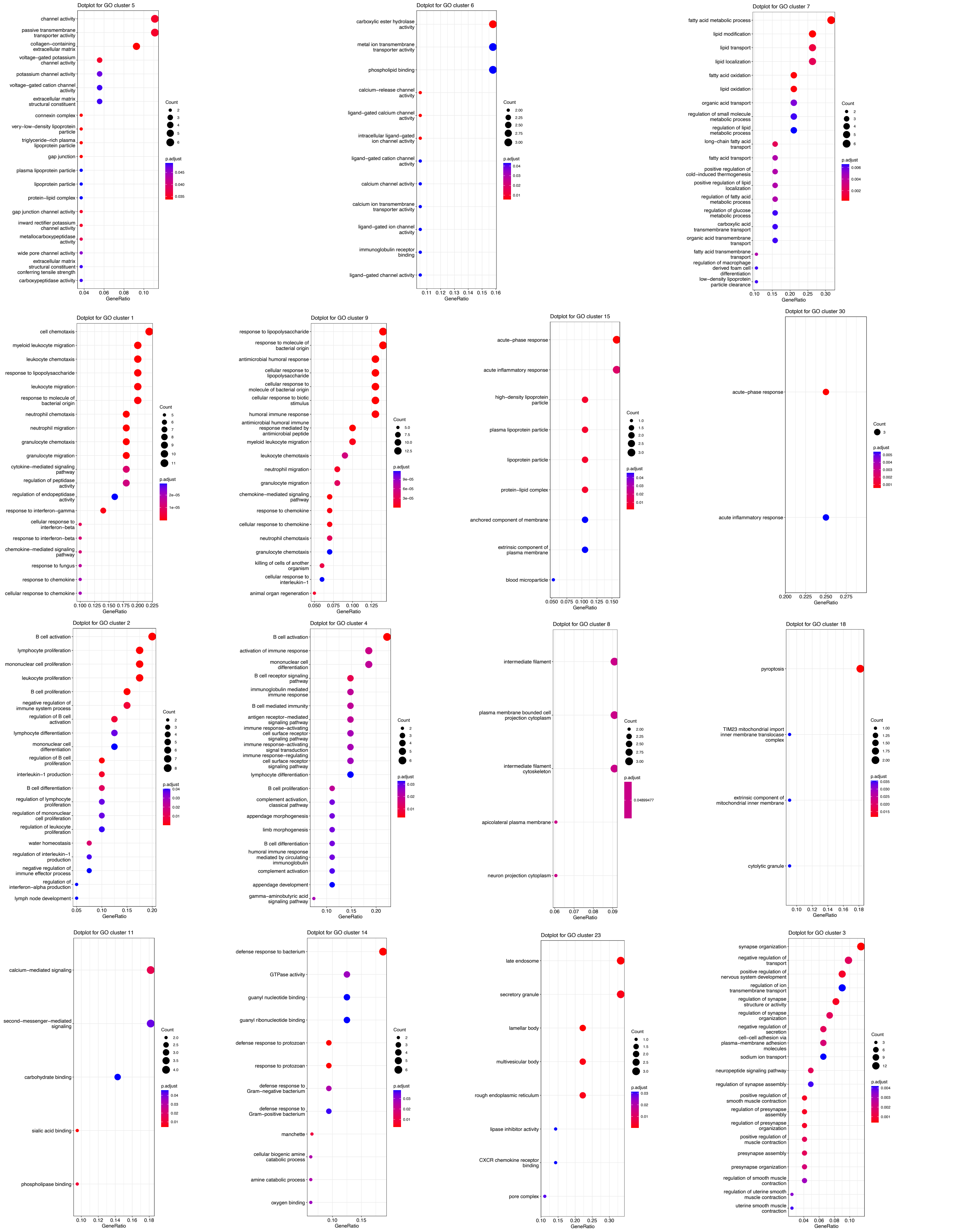

Figure S9. Decrease in colonic homeostasis genes and loss of colonocytes that are not repleshined in aged infected colons.

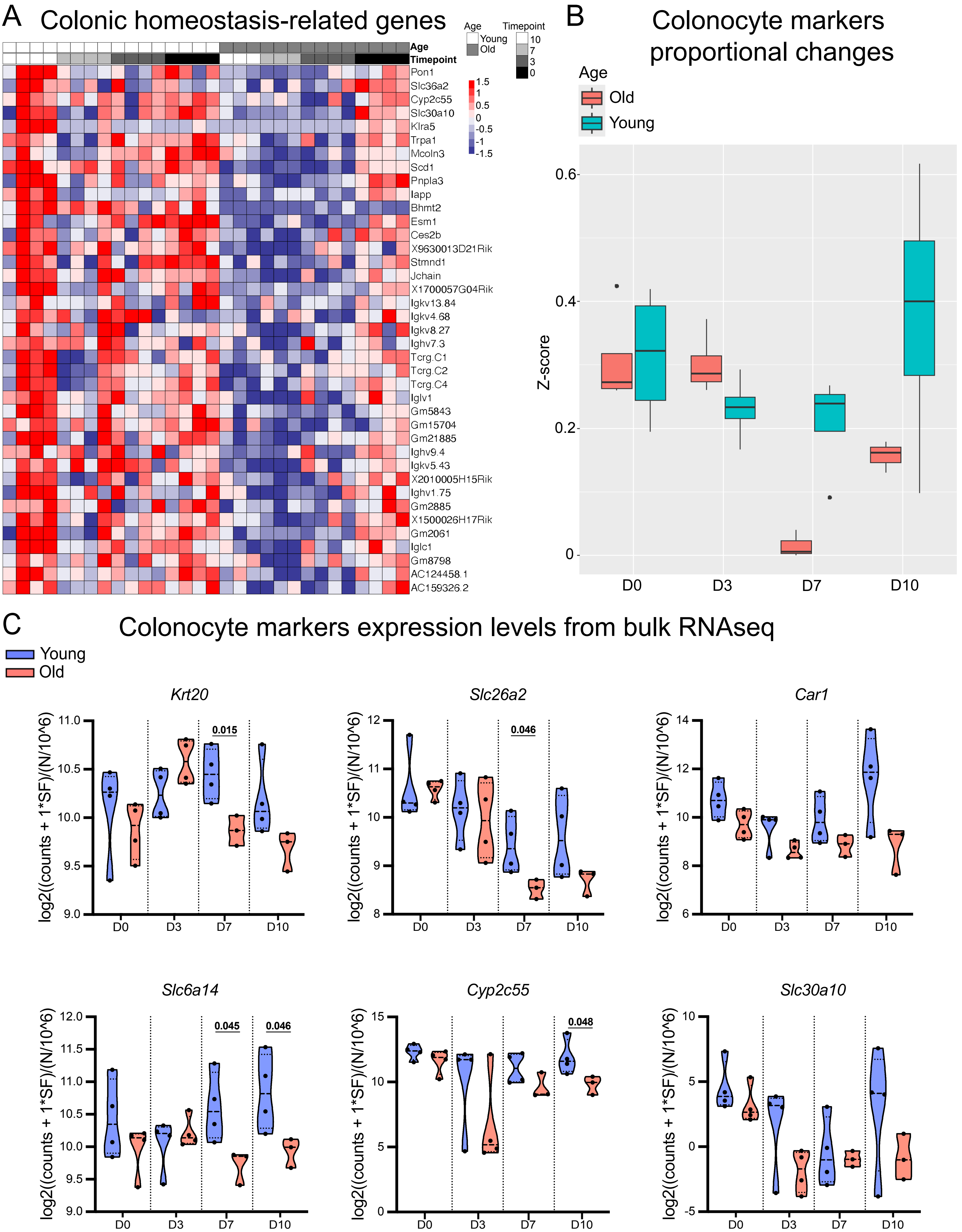

Figure S10. Genes belonging to the inflammation-related cluster.

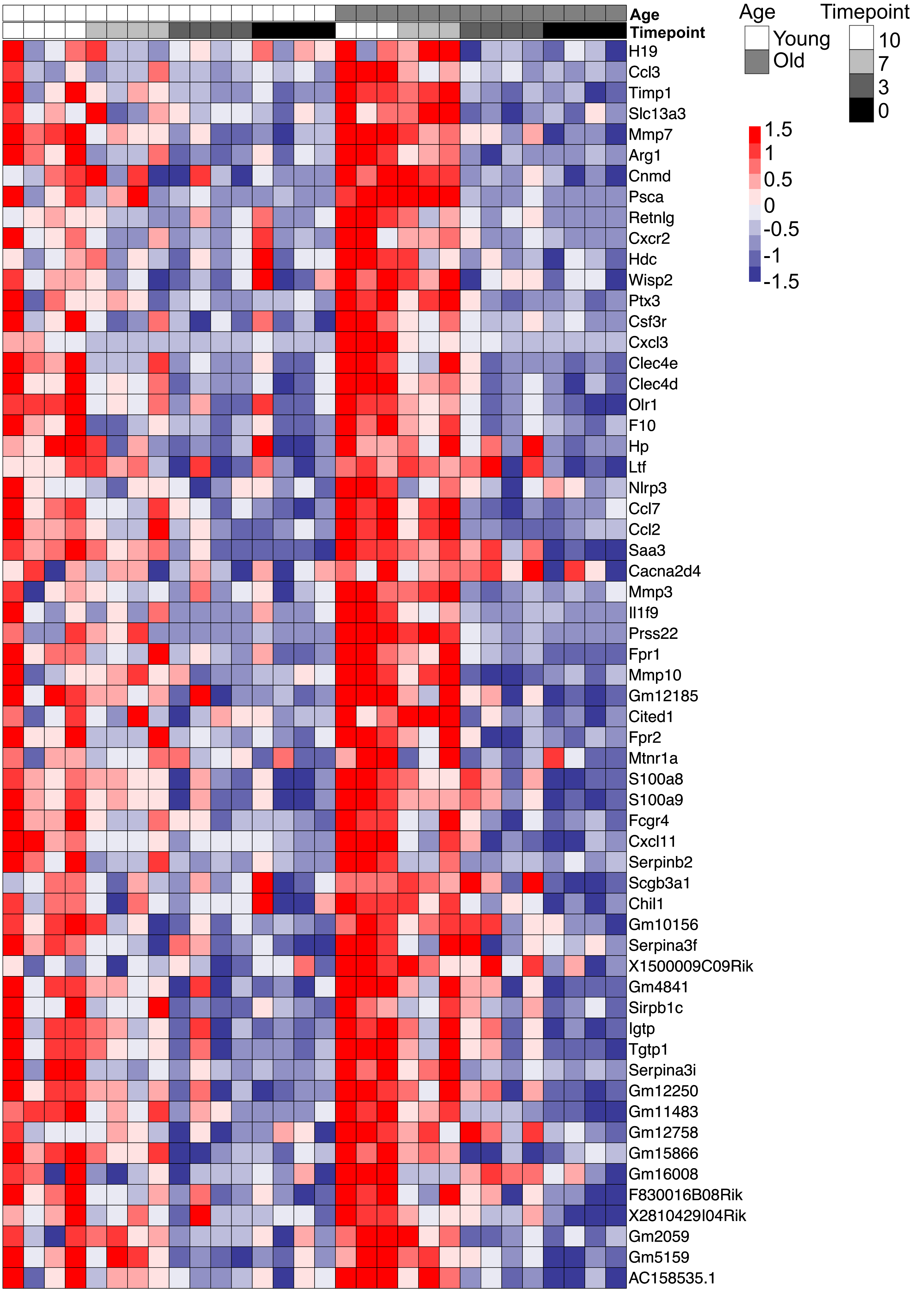

Figure S11. Frequency of IFNγ^{+} immune cells in the colon of *C. rodentium*-infected mice.

A

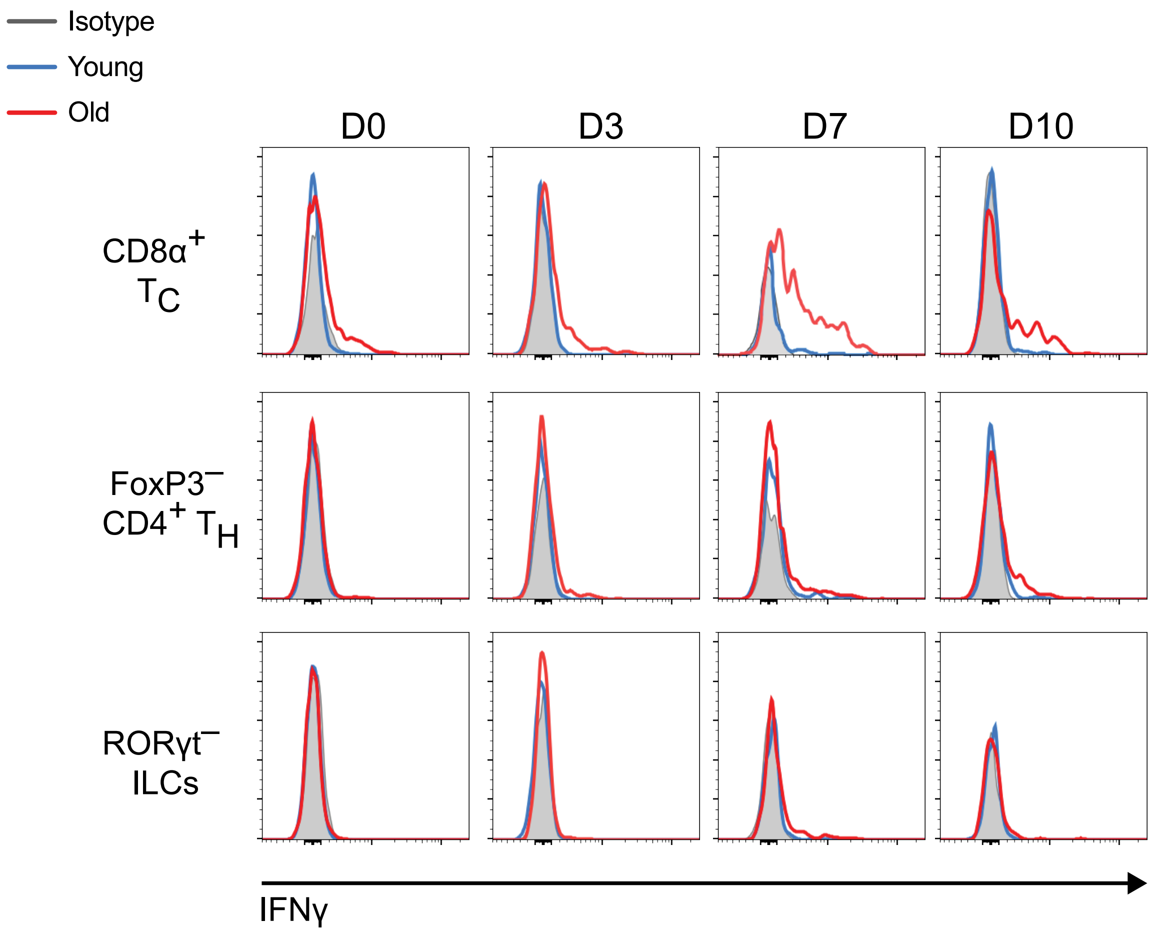

Figure S12. Aging enhances epithelial sensitivity to IFNγ and vulnerability to cell death.

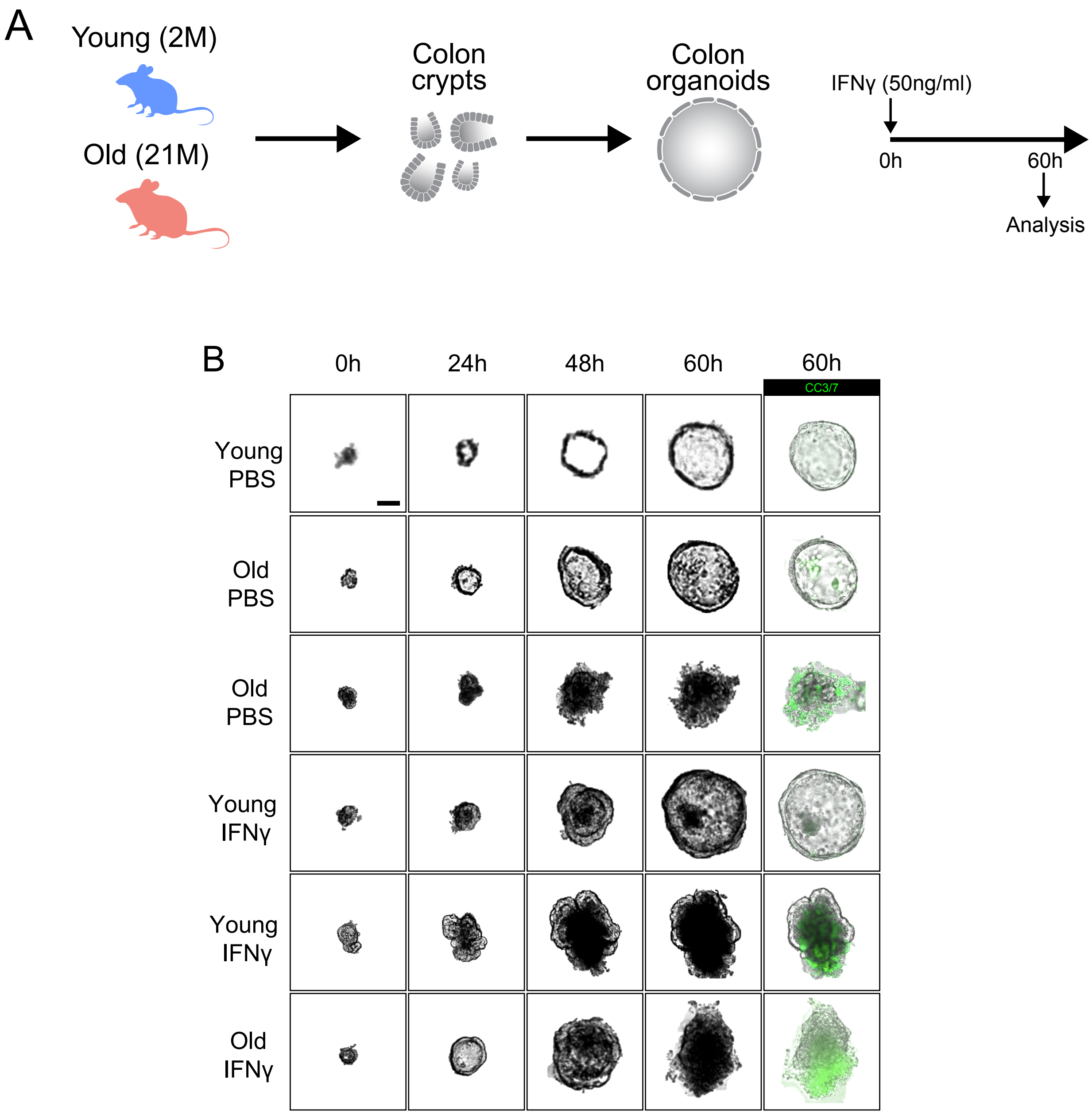

Figure S13. Heatmaps of gene expression for genes in the fetal signature

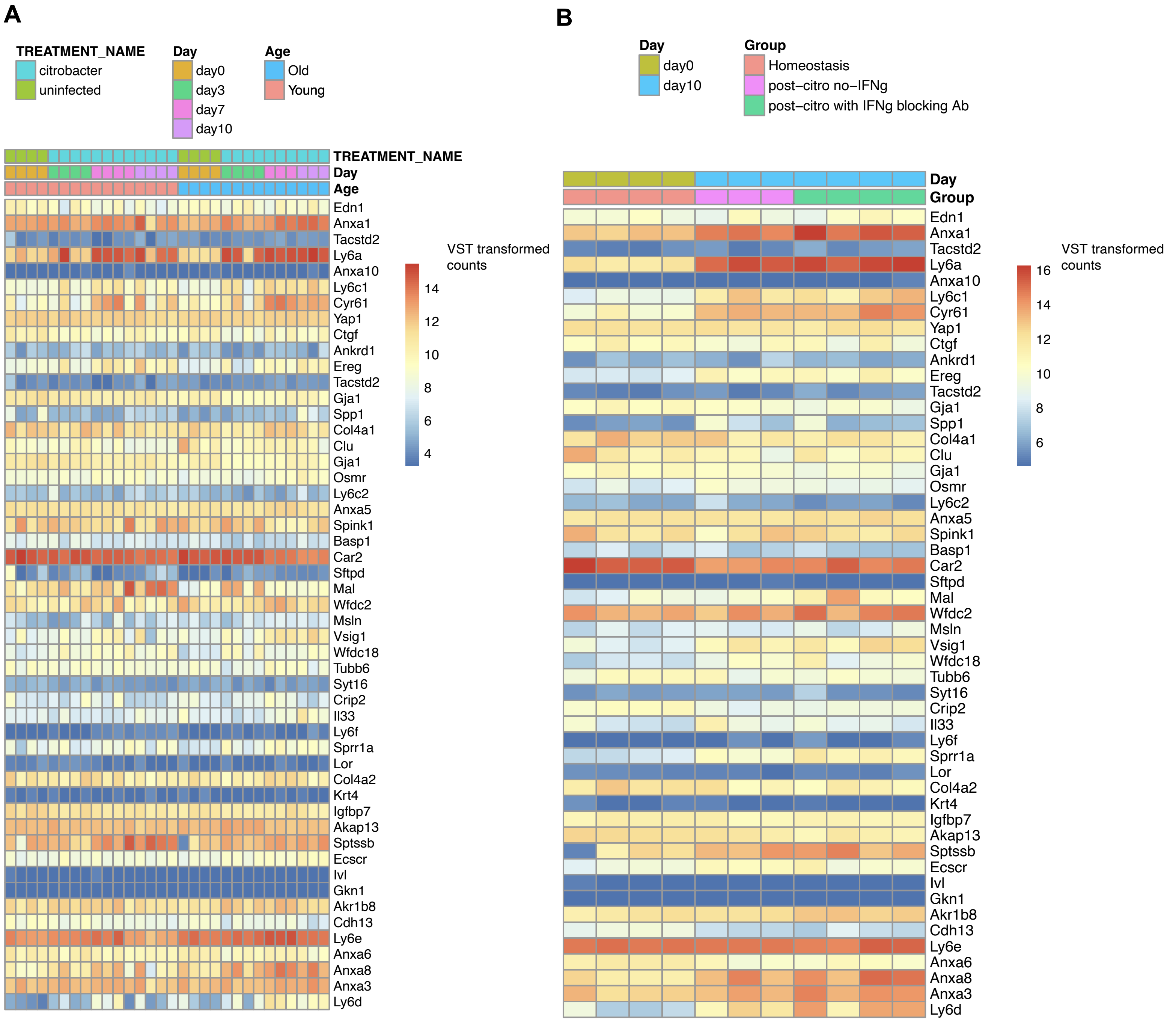

Figure S14. The impact of anti-IFNγ antibody on citrobacter infection phenotypes

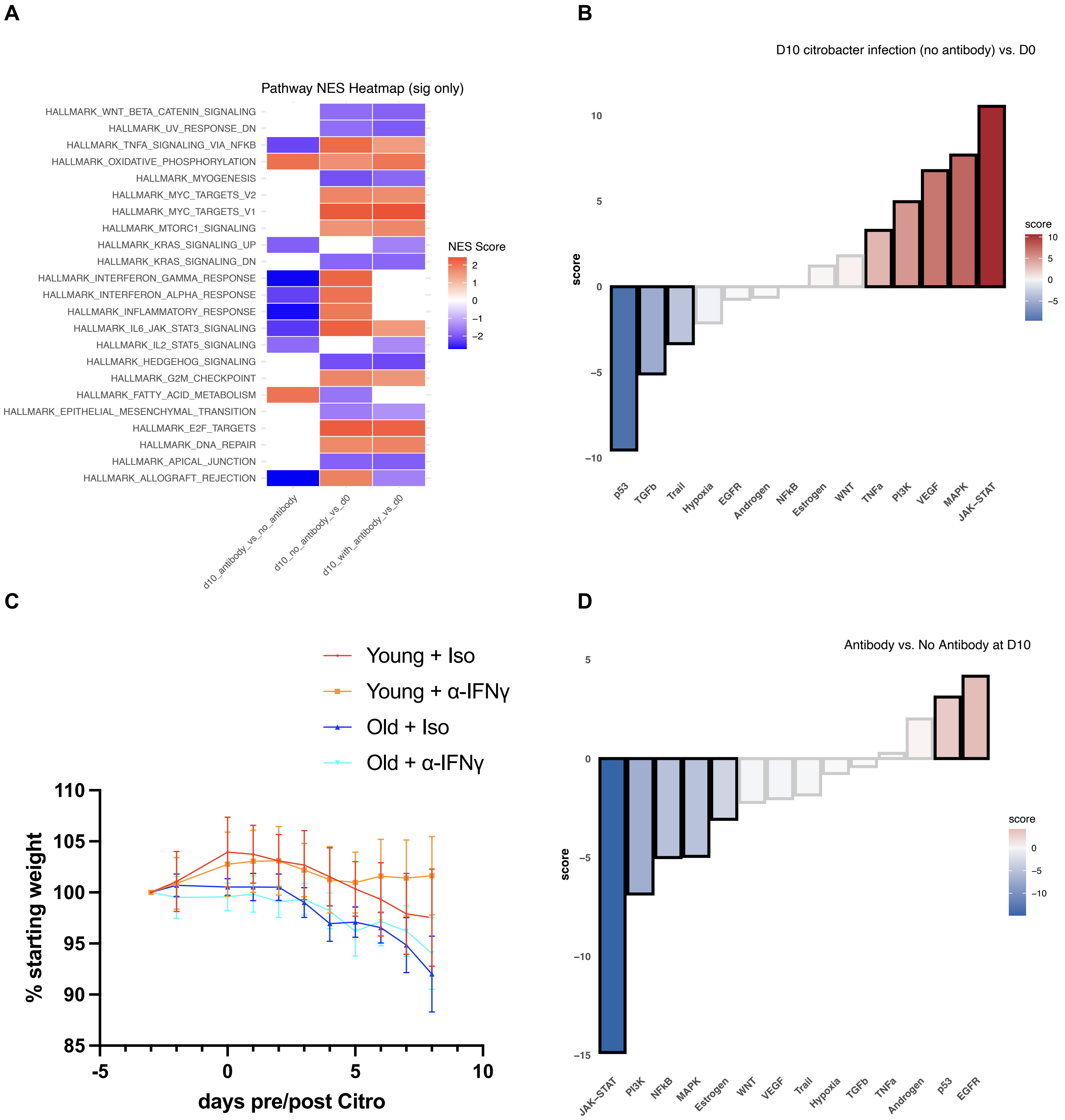

Supplement: Supplementary file 1 — Figure S1: Markers used for annotation of the colon epithelial and immune cell types with epithelial cell type refined annotations and differential expression (A) Dotplot depicting the expression levels of the markers used for annotation of the colon epithelial cell types. (B) Dotplot depicting the expression levels of the markers used for annotation of the colon immune cell types. (C) Sub‐clustering of stem‐cell like neighborhoods from the colon with per‐sample level integration performed with Harmony. (D) Milo analysis of sub‐clustered cells in (C). While very few neighborhoods reach nominal significance in a model containing a “study” effect, the Ascl2‐low enriched cluster 9 shows a clear shift in estimated log FC in old colon relative to young. Colored dots represent significantly differentially abundant cell neighborhoods with a spatially adjusted FDR of < 0.1. (E) MA plot showing the average log2FC in gene expression between aged and young colon epithelium. Points shown in red pass an FDR threshold of ≤ 0.1. Figure S2: Evidence for similar bacterial clearance rates in old versus young colons (A) Log transformed read count values for the expression of core bacterial response genes in citrobacter infected old animals versus controls during the anti‐IFNγ antibody experiment. (B) Log transformed read count values for the expression of core bacterial response genes in a citrobacter infection time course experiment in young and old animals showing no significant differences in gene expression between old and young animals following infection. (C) Bacterial CFU in the anti‐IFNγ antibody experiment covering both old and young animals during a time course experiment showing essentially unchanged infection levels in old versus young animals across the time course. Figure S3: Analysis of Cleaved Caspase‐3 (CC3) expression in young and aged colonic epithelium 7 days after C. rodentium infection. (A) Representative immunofluorescence images showing Cleaved Caspase‐3 (CC3) [file ACEL-25-e70495-s002.pdf]
